# Photoacoustic Imaging of Vascular Structure After Breast Reconstruction with Autologous Fat Grafting: A Pilot Study

**DOI:** 10.3390/jcm15031272

**Published:** 2026-02-05

**Authors:** Yui Tsunoda, Mayu Muto, Minami Noto, Toshihiko Satake

**Affiliations:** 1 Lala Breast Reconstruction Clinic Yokohama, Yokohama Kokusai Building 9F, 3-28 Onoecho, Naka-ku, Yokohama 231-0015, Japan; ytsunoda@lala-brc.jp (Y.T.); mayumuto@lala-brc.jp (M.M.); 2 Department of Plastic, Reconstructive and Aesthetic Surgery, Faculty of Medicine, University of Toyama, 2630 Sugitani, Toyama 930-0194, Japan; minami@med.u-toyama.ac.jp

**Keywords:** breast reconstruction, autologous fat grafting, photoacoustic imaging, neovascularization, vascular density, vascular remodeling, vessel diameter

## Abstract

**Background/Objectives:** Autologous fat grafting (AFG) is widely used in breast reconstruction; however, graft retention remains unpredictable due to recipient-bed variability. Photoacoustic imaging (PAI) is a contrast-free, noninvasive modality enabling visualization of vascular structures in detail. This study used PAI to visualize and quantitatively assess neovascularization and vascular structure in breasts reconstructed with AFG. **Methods:** In this retrospective, cross-sectional study, data from eight patients who underwent PAI of both reconstructed and contralateral breasts at least three months after their final AFG procedure for total breast reconstruction were used. Excluding the nipple–areola complex and skin markings, four 3 × 3 cm regions of interest (one per quadrant) were selected in the periareolar region. Vascular density in terms of depth from the skin surface was analyzed in five cases with adequate contact between the device and the skin. Visible vessel diameters within the regions of interest were manually measured and categorized as small, medium, or large to assess distribution patterns. **Results:** PAI successfully enabled visualization of vascular structures on the reconstructed side in all cases, even at depths greater than 10 mm. In five cases, vascular density in the superficial layer (0–2.5 mm) was higher on the reconstructed side than on the contralateral side. A longer postoperative interval was associated with a higher proportion of small vessels and fewer large vessels. **Conclusions:** PAI enabled noninvasive visualization of vascular structures consistent with neovascularization on the reconstructed side after AFG. Temporal changes in vessel diameter distribution suggest ongoing vascular remodeling, supporting the potential utility of PAI in assessing vascular structural changes in grafted tissue over time.

## 1. Introduction

Breast reconstruction, which is broadly categorized into prosthetic reconstruction and autologous tissue reconstruction, has been associated with an improved quality of life for patients with breast cancer [1]. Autologous techniques include flap reconstruction and autologous fat grafting (AFG). After the introduction of Coleman’s concept of structural fat grafting, AFG has gained wide acceptance as a reliable method [2]. A recent bibliometric analysis of 3404 publications worldwide identified fat grafting as one of the most actively researched topics in postmastectomy breast reconstruction over the past decade [3]. Total breast reconstruction with AFG is associated with several advantages, including the fact that it requires only small incisions and is a minimally invasive technique, and that reconstruction of a breast with natural texture and appearance can be achieved without implants. However, this approach also has some disadvantages, including the need for multiple procedures and the risk of fat necrosis or calcifications that can complicate radiological assessment. Nevertheless, AFG has been shown to be both safe and effective when performed appropriately [4].

The variable and unpredictable retention rate associated with AFG is clinically challenging. In breast reconstruction following breast cancer surgery, fat graft retention varies widely among patients, as final volume retention depends on the recipient-site microenvironment (e.g., vascularity/oxygenation), in addition to graft size, technique, and postoperative care. Fat is transplanted without its own blood supply, necessitating revascularization (angiogenesis) from the surrounding tissues for graft survival [5]. Therefore, assessing angiogenesis and vascularity on the reconstructed side is imperative for improving fat graft survival and predicting outcomes.

Photoacoustic imaging (PAI) is a promising novel evaluation modality. In this technique, after irradiating the tissue with pulsed laser light, components such as hemoglobin absorb optical energy and undergo thermoelastic expansion. Detection of the resulting ultrasonic waves, known as photoacoustic signals, enables the reconstruction of high-resolution three-dimensional images, particularly of the vasculature, without requiring contrast agents. In addition, its noninvasive nature facilitates longitudinal monitoring [6].

To date, PAI research in the field of breast cancer has primarily focused on detecting tumor vasculature [6,7] and visualizing blood vessels in free flaps [8]. However, to the best of our knowledge, PAI has not been used for evaluating vascular structures in breasts reconstructed with AFG. This study aimed to use PAI for analyzing vascular structures following total breast reconstruction with AFG, and for exploring the visualization and quantitative assessment of neovascularization.

## 2. Materials and Methods

This exploratory, retrospective, and cross-sectional study was performed on a small cohort of patients who had undergone total breast reconstruction with AFG for unilateral breast cancer at either the University of Toyama or its affiliated facility, Lala Breast Reconstruction Clinic Yokohama, and were at least three months post-surgery.

As the approved protocol permitted including data from up to six months prior to the approval date, we retrospectively analyzed patients who had undergone PAI between 31 March 2024 and 29 August 2024. PAI was performed at the facility of our research collaborator, Luxonus Inc. (Kawasaki, Japan); therefore, the analyzed dataset consisted of patients from the nearby affiliated clinic.

The inclusion criteria were age 18 years or older and voluntary provision of signed informed consent. The exclusion criteria were (1) pregnancy or breastfeeding, (2) current use of photosensitizing agents, (3) presence of a cardiac pacemaker or implantable cardioverter–defibrillator (ICD), and (4) conditions deemed unsuitable by the attending physician, such as difficulty in communication or inability to maintain the imaging position. Based on these criteria, a total of eight patients were included in the final analysis.

This study was approved by the Ethics Review Committee of the University of Toyama (Approval Number: R2024114; Approval Date: 24 September 2024) and was conducted in accordance with the Declaration of Helsinki. Written informed consent was obtained from all participants.

**Figure 1:**
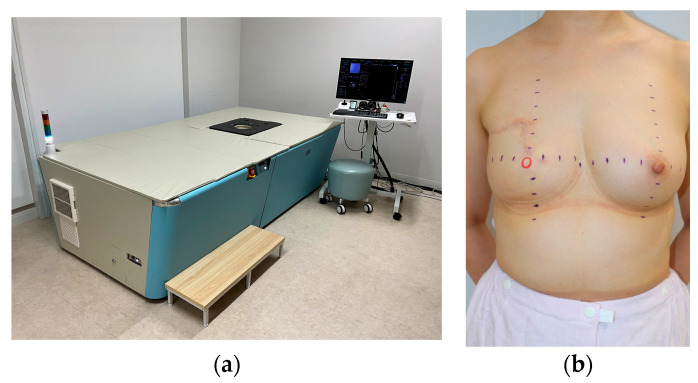
(**a**) The LUB-0 photoacoustic imaging system. Imaging is performed in the prone position; (**b**) photograph depicting the skin markings. Purple markings were applied at 2 cm intervals extending cranially, caudally, medially, and laterally from the nipple. In cases of nipple excision, a red circular marking was made to indicate the estimated nipple position. The purple markings are visible in the photoacoustic images, whereas the red markings are not visible.

Photoacoustic imaging was performed using the LUB-0 system (Luxonus, Kawasaki, Japan) (Figure 1a), which enabled label-free imaging, involved no radiation exposure, and allowed for the acquisition of high-resolution vascular images [8,9]. In addition, the calculation of the S-factor, a hemoglobin oxygenation ratio, was possible due to the dual-wavelength excitation (797 nm and 756 nm), which enabled differentiation between adjacent arteries and veins [10].

Purple markings were made at 2 cm intervals extending from the nipple to the breast periphery cranially, caudally, medially, and laterally to ensure the reproducibility of the imaging position. In cases of nipple excision, the estimated nipple position was marked with a red circular marking (Figure 1b). The purple markings are visible in the images, whereas the red markings are not visible. Imaging was performed on both sides (the reconstructed and contralateral sides).

**Figure 2:**
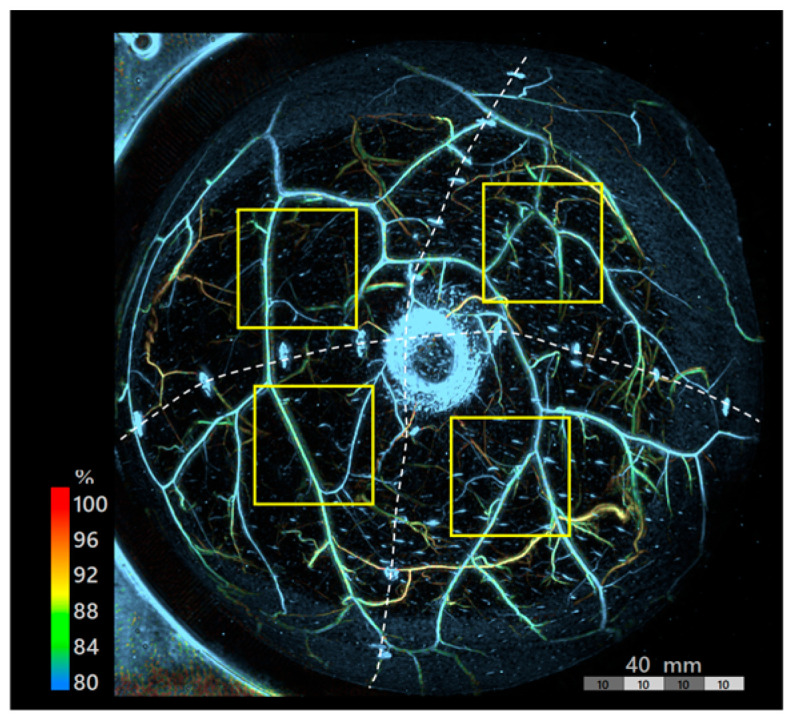
Representative image depicting ROI selection (Case 1). The image was divided into four quadrants (white dashed lines) based on the skin markings. A 3 × 3 cm ROI (yellow solid line) was selected just outside the border of the nipple-areola complex to avoid pigmentation and markings.

The primary endpoints were vascular density and vessel diameter. Dedicated software (PAT Viewer Ver. 6.53, Luxonus, Kawasaki, Japan) was used for performing the image analysis. The images were divided into four quadrants based on the skin markings to minimize regional variability. A 3 × 3 cm region of interest (ROI) was selected in each quadrant just outside the border of the nipple–areola complex (or estimated position) to avoid areolar pigmentation and skin markings (Figure 2).

Python (version 3.11.4) was used to perform vascular density analysis. Each ROI was segmented into eight depth layers (0–2.5, 2.5–5, 5–7.5, 7.5–10, 10–12.5, 12.5–15, 15–17.5, and 17.5–20 mm) at intervals of 2.5 mm from the skin surface. Images were converted to grayscale (0–255) and binarized using a threshold of 75. This threshold was empirically determined by testing several values (50, 75, and 100) on pilot images from a subset of cases and selecting the value that best delineated vessels while minimizing background noise. A fixed threshold, rather than adaptive thresholding, was used to ensure objectivity and reproducibility of the analysis across all ROIs and cases, thereby enabling an unbiased comparison between the reconstructed and contralateral sides. Vascular density (%) was calculated as the ratio of pixels above the threshold to the total number of pixels in each layer. Due to issues related to the definition of depth from the skin surface, three cases (Cases 2, 5, 8) were excluded from the vascular density analysis. In these cases, there was a discrepancy between the true depth from the skin surface and the analytical depth arising from insufficient contact between the imaging device and the skin surface.

Vessel diameters were manually measured within each ROI by a single operator. Measurements were taken at clearly visible locations where vessels appeared relatively straight. For bifurcating vessels, measurements were taken proximal and distal to the branch point, avoiding the widened bifurcation region. For unbranched vessels, a single measurement was taken at the straightest segment. Data from the contralateral side were used to calculate quartile values across all cases (n = 2008 measurement points). The resulting values were as follows: first quartile, 0.29 mm; median, 0.37 mm; and third quartile, 0.50 mm. Based on these values, 0.3 mm and 0.5 mm were selected as rounded cutoff thresholds for classifying vessels into three categories: small (0.08–0.3 mm), medium (0.3–0.5 mm), and large (0.5–1.3 mm). Specifically, vessel diameters of exactly 0.3 mm and 0.5 mm were classified into the medium group.

Since this exploratory study was performed on a small cohort (n = 8), the results are primarily reported using descriptive statistics. Depending on the data distribution, continuous variables are reported as either mean ± standard deviation or median (range).

AI-assisted tools were used for translation and English language editing to improve the readability of the manuscript.

## 3. Results

PAI was performed on all eight participants who had undergone total breast reconstruction using AFG (Table 1). All patients underwent delayed breast reconstruction with AFG alone, without flaps or implants. The median age of the patients was 54.5 years (range, 43–67 years), and the median body mass index (BMI) was 22.3 kg/m^2^ (range: 20.0–27.9 kg/m^2^). The median number of AFG sessions was 3.5 (range: 2–7), and the median interval from the final surgery to imaging was 15 months (range: 3–30 months).

Six patients underwent two-stage reconstruction: a temporary tissue expander was placed at the time of mastectomy, and AFG was subsequently initiated with the expander in situ. The tissue expander was removed during one of the subsequent AFG sessions. The remaining two patients underwent direct AFG without prior tissue expander placement. Regarding adjuvant therapies, one patient (Case 6) received radiation therapy before AFG initiation. Details of the AFG procedures, including whether cultured adipose-derived stem cells (ASCs) were added, are summarized in Table 1. No postoperative complications were reported.

**Figure 3:**
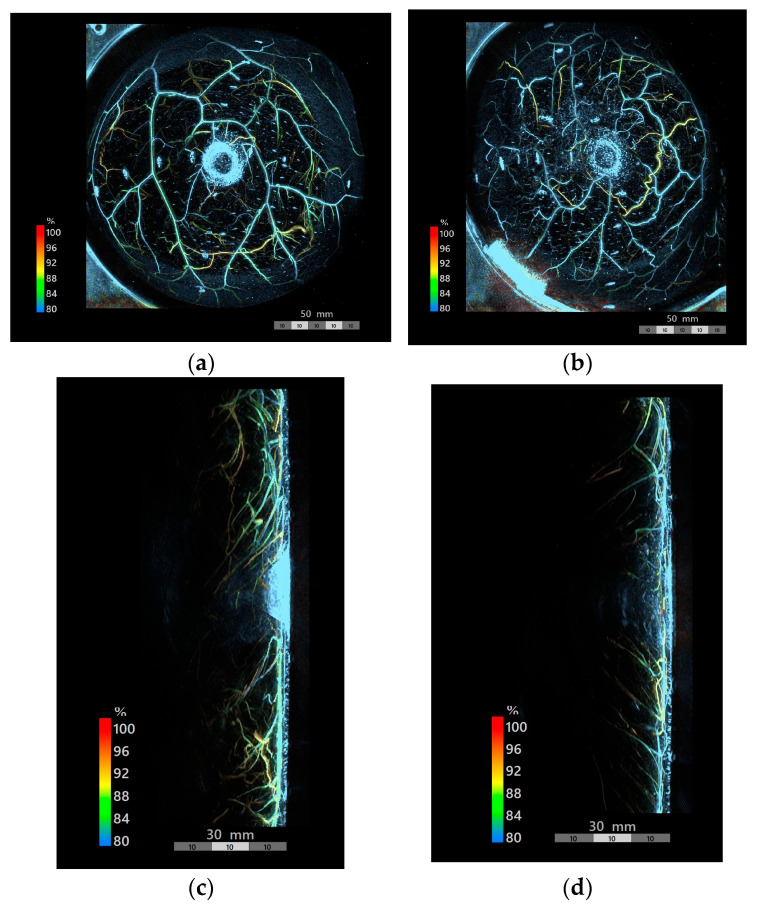
PAI images of Case 1: (**a**) contralateral side; (**b**) reconstructed side; (**c**,**d**) sagittal views of contralateral and reconstructed sides, respectively. In the sagittal view, depth increases from right (superficial, near the skin surface) to left (deep layer).

PAI was performed to visualize vascular structures in both breasts in all cases (Figure 3a,b). Vascular structures were observed throughout. Sagittal views revealed numerous superficial vessels extending to approximately 20 mm depth on the contralateral side (Figure 3c) and exceeding 10 mm depth on the reconstructed side (Figure 3d).

**Figure 4:**
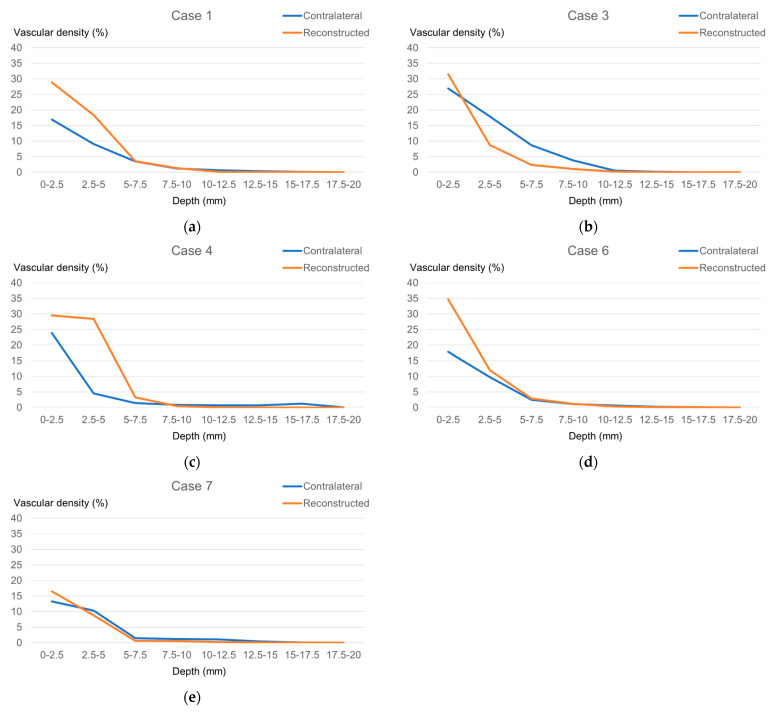
Depth profiles of vascular density in five cases with accurate depth referencing from the skin surface. (**a**) Case 1; (**b**) Case 3; (**c**) Case 4; (**d**) Case 6; (**e**) Case 7. (Orange line: reconstructed side; blue line: contralateral side).

We segmented the 0–20 mm depth range into eight layers for both sides for analyzing vascular density. As previously mentioned in Section 2, only the five cases in which depth could be measured accurately from the skin surface were included in the analysis (Figure 4a–e). Vascular density decreased with increasing depth on both sides. A consistent trend was observed across all cases at the most superficial layer (0–2.5 mm): vascular density was higher in the reconstructed side (28.2 ± 6.8%) compared with the contralateral side (19.8 ± 5.5%).

**Figure 5:**
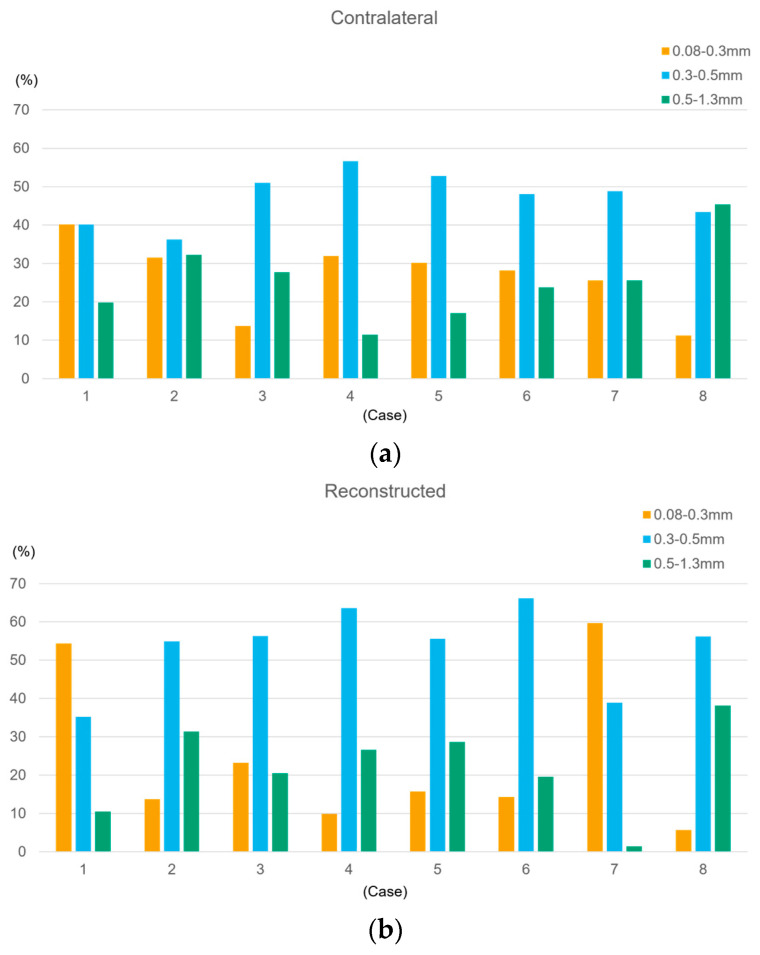
Distribution of vessel diameter categories. Vessels were classified into three groups based on quartiles derived from the contralateral-side data: small (0.08–0.3 mm; yellow bars), medium (0.3–0.5 mm; blue bars), and large (0.5–1.3 mm; green bars): (**a**) proportions of vessels in the contralateral side; (**b**) proportions of vessels in the reconstructed side.

To characterize the distribution of vessel diameters, the data (n = 2008 measurement points) were classified into three categories: small (0.08–0.3 mm), medium (0.3–0.5 mm), and large (0.5–1.3 mm). In each case, the proportion of each category was calculated for both sides (Figure 5a,b). While medium vessels were predominant on the reconstructed side in most cases, the proportion of small vessels was notably high in Case 1 (54.3%) and Case 7 (59.7%). The intervals from the final surgery were 26 months and 30 months in Cases 1 and 7, respectively.

**Figure 6:**
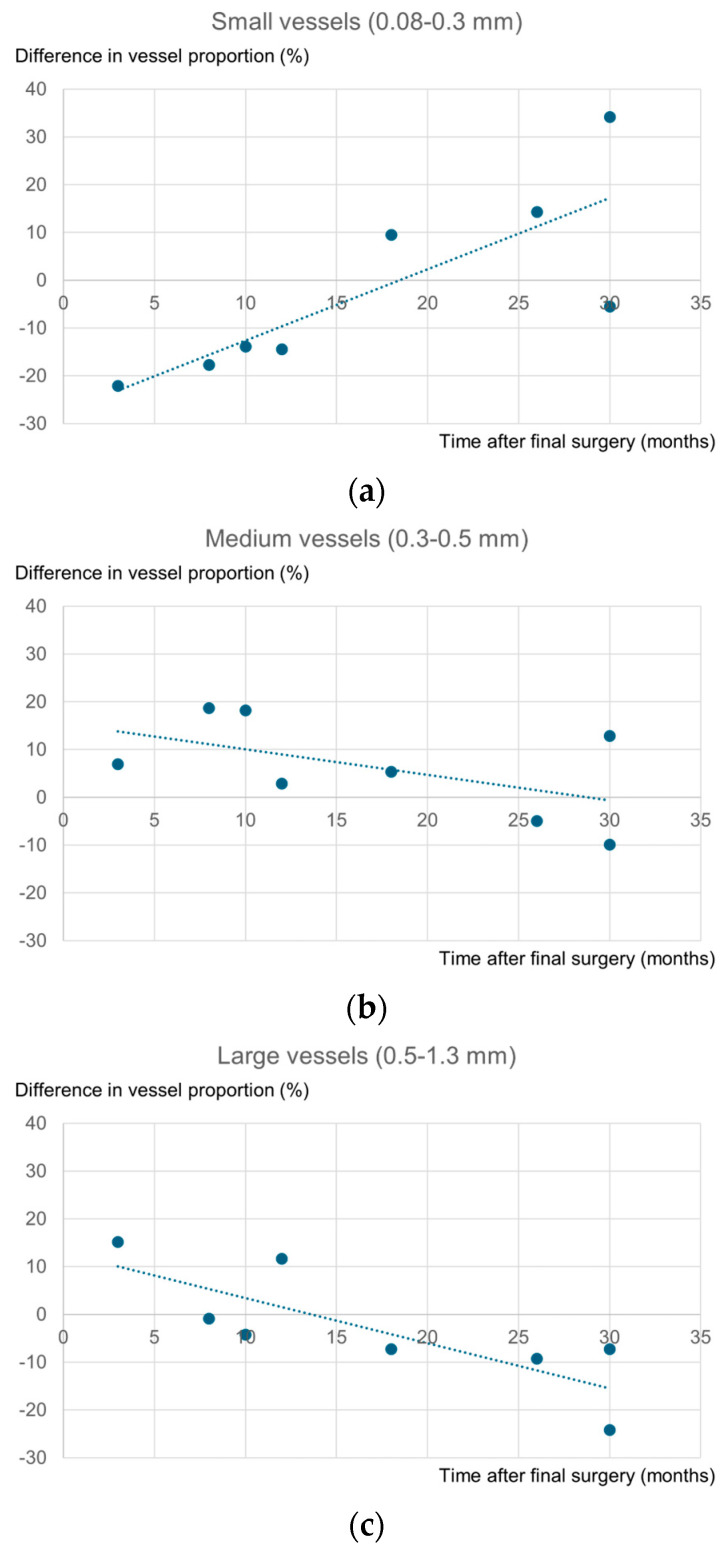
Relationship between the interval from the final surgery and the difference in vessel proportions. The vertical axis represents the difference in proportions (reconstructed minus contralateral). The dotted line represents the trend line: (**a**) small vessels (0.08–0.3 mm); (**b**) medium vessels (0.3–0.5 mm); (**c**) large vessels (0.5–1.3 mm).

Based on these results, to examine the relationship between the interval from the final surgery and changes in vascular diameter distribution, we plotted the difference in proportions between sides (reconstructed minus contralateral) against the postoperative interval for all cases (Figure 6a–c). The analysis suggested that the proportion of small vessels tended to increase with longer intervals from the final surgery, whereas the proportion of large vessels tended to decrease. In contrast, no clear trend was observed for the proportion of medium vessels in relation to the interval from the final surgery, likely due to data variability.

Given the limited sample size of this study, these findings should be considered exploratory and interpreted with caution.

## 4. Discussion

In this study, we employed photoacoustic imaging (PAI) to assess the vascular structure of breasts reconstructed with AFG. PAI successfully visualized high-resolution vasculature extending to deep tissue layers, thereby supporting its value as a noninvasive imaging modality. Quantitative analysis indicated that vascular density in the superficial layer (0–2.5 mm) was higher on the reconstructed side than on the contralateral side. Furthermore, an analysis of vessel diameter in relation to the postoperative period showed a trend toward an increased proportion of small vessels and a decreased proportion of large vessels over time, suggesting progressive changes in the vascular structure.

In the sagittal PAI images, blood vessels extending from the superficial to the deep layers were clearly visualized. Based on our clinical experience with preoperative ultrasound assessments, the residual subcutaneous tissue thickness following total mastectomy in Japanese patients is typically less than 10 mm. This anatomical observation is supported by Larson et al. [11], who reported a median subcutaneous tissue thickness of 10 mm from the dermis to the mammary gland parenchyma, and by Dassoulas et al. [12], whose MRI and cadaveric studies demonstrated an average thickness of 5.5–6.6 mm from the skin to the superficial fascia, thereby corroborating our findings. This indicates that the blood vessels observed at depths greater than 10 mm are unlikely to have originated from the residual native tissue. Instead, they potentially represent neovascularization within the engrafted adipose tissue, consistent with the mechanism of recipient-derived vascular ingrowth demonstrated in murine models by Dong et al. [13]. Our findings suggest that PAI observations align with these preclinical data, thereby indicating the feasibility of visualizing neovascularization in AFG in a noninvasive manner.

Regarding the quantitative analysis of vascular density, both the reconstructed and contralateral sides exhibited a depth-dependent decrease in vascular density. There are two possible explanations for this observation. First, from an anatomical perspective, the subcutaneous adipose layer has a lower vascular density compared to the deep dermis. Second, the technical limitations of PAI must be considered. The excitation light undergoes scattering and attenuation in deeper tissues, leading to reduced signal intensity and diminished sensitivity for vessel detection. This phenomenon aligns with previous studies that have reported signal attenuation and difficulty visualizing deep tissue structures in breast PAI [6,7]. Taken together, these factors could have contributed to the lower vascular density values calculated in the deeper layers.

The vascular density in the superficial layer was greater on the reconstructed side compared with the contralateral side. This observation is likely associated with the pattern of angiogenesis induced by AFG. Because the subjects in this study underwent reconstruction after total mastectomy, in which the mammary gland tissue is absent, AFG was performed in multiple layers: subcutaneous, intramuscular (pectoralis major), and retromuscular. Importantly, fat was also injected directly beneath the skin, placing the grafted adipose tissue in close contact with the highly vascularized skin. Dong et al. demonstrated that recipient-derived vessels undergo angiogenesis and grow into the graft [13]. Therefore, it can be assumed that the increased vascular density observed in the most superficial layer in this study reflects enhanced vascular branching into the graft from the pre-existing cutaneous vascular network. It should be noted that the preserved skin and subcutaneous tissue after mastectomy experience transient ischemia, which may lead to changes similar to the so-called delay phenomenon. In this phenomenon, early vasodilation occurs, but long-term studies have shown that despite easy vasodilation occurring in this phenomenon, redundant vessels are pruned according to perfusion demand, leaving only dilated choke vessels [14]. Because the cases in this study were evaluated long after breast cancer surgery, it is unlikely that any increase in vascular density attributable to the delay phenomenon persisted. Therefore, the elevated superficial vascular density observed here is more plausibly explained by angiogenesis induced by AFG rather than by the lasting effects of the delay phenomenon.

Regarding the vascular diameter analysis, a longer interval since the final surgery correlated with an increased proportion of small vessels and a decreased proportion of large vessels. This trend likely reflects the vascular remodeling process associated with angiogenesis. Angiogenesis begins with the dilation of existing vessels, followed by sprouting to form new branches. It is well established that this transiently hyperplastic vascular network eventually undergoes pruning of redundant vessels, reorganizing into a finer, stable, and mature microvascular structure [15]. In the context of AFG, ischemia and hypoxia immediately after transplantation elicit an angiogenic response [16], and capillary ingrowth into the graft begins as early as 1 week and peaks around 4 weeks after transplantation [17]. Revascularization is driven by the ingrowth of recipient-derived vessels [13], while ASCs within the regenerating tissue contribute to both angiogenesis and tissue repair [5]. Transplanted adipose tissue has been reported to mature over a period of 1–3 months [18]. As the tissue matures and oxygenation stabilizes, vessels that were initially dilated due to ischemia and inflammation return to normal caliber, while redundant vessels are removed through pruning. The observed shift consisted of a reduction in large vessels and an increase in small vessels. This pattern suggests a remodeling process characterized by the normalization of previously dilated vessels and reorganization into a more efficient microvascular network.

This study has several limitations. First, this study included only eight patients and adopted a retrospective, cross-sectional design, resulting in limited statistical power. The absence of preoperative baseline PAI data precludes distinguishing whether the vascular changes observed in this study represent AFG-induced neovascularization or other tissue remodeling processes, thereby limiting causal inference. Furthermore, confounding factors arising from patient heterogeneity could not be adequately adjusted for. Specifically, factors such as the presence or absence of radiation therapy prior to AFG initiation, the addition of cultured adipose-derived stem cells (ASCs), and the use and subsequent removal of tissue expanders may have influenced vascular remodeling patterns; however, the small sample size prevents us from isolating the effects of these factors. Second, the use of the contralateral side as a control has limitations. It has not undergone surgical trauma and retains mammary gland tissue, both of which may introduce differences compared with the reconstructed side. An ideal comparison would include preoperative baseline PAI of the ipsilateral breast, which was not feasible in this retrospective study. Third, there are technical challenges in image analysis. While a fixed threshold ensures objectivity, it may not account for local variations in signal intensity. Light attenuation in deeper tissues is an inherent limitation of PAI, making it difficult to distinguish whether the depth-dependent decrease in vascular density reflects true anatomical differences or signal loss. Evaluating cases with thinner tissue could help clarify this distinction, as light attenuation would be less influential. Future studies should also consider analysis strategies that reduce the impact of depth-related signal loss, such as within-subject relative comparisons. Additionally, three cases were excluded from depth-specific analysis due to insufficient contact between the imaging device and the skin surface, potentially introducing selection bias. Fourth, vessel diameters were manually measured by a single operator, which may introduce operator-dependent variability. Intra-observer and inter-observer reliability were not assessed. Moreover, microvessels below the resolution limit of the PAI system may have been systematically underestimated or not detected, potentially affecting the reported proportion of small vessels.

Despite these limitations, this study demonstrates the feasibility of noninvasively visualizing and quantifying vascular changes in breasts reconstructed with AFG using PAI. Future studies should employ prospective, longitudinal designs with PAI assessments at multiple time points before and after surgery to directly quantify AFG-induced vascular changes. Larger sample sizes would enable stratified analyses of how patient-specific factors, including radiation therapy, ASC supplementation, and tissue expander use, affect vascular remodeling. Additionally, automation of vessel diameter measurements and image analysis is desirable to reduce operator bias and improve objectivity.

Furthermore, investigating correlations between PAI metrics and clinical outcomes (fat graft retention, complication rates, patient satisfaction) is essential to establish PAI as a clinical tool. PAI may provide value at multiple stages of AFG management. Preoperatively, if baseline PAI can predict poor graft survival, surgeons could employ strategies such as external tissue expanders to optimize recipient bed vascularity. Postoperatively, serial PAI monitoring may enable an objective assessment of vascular remodeling: the shift from large to small vessels and stabilization of vascular density may indicate maturation of the vascular network, providing objective criteria for determining the optimal timing of subsequent AFG sessions.

## 5. Conclusions

In conclusion, this study demonstrated the potential of PAI for visualizing neovascularization and assessing vascular structural changes following AFG. PAI successfully depicted vascular structures within the grafted tissue on the reconstructed side, with quantitative analysis showing higher superficial layer vascular density compared with the contralateral side. In addition, the increase in small vessels and the decrease in large vessels over the postoperative course suggest ongoing vascular remodeling within the transplanted adipose tissue. Future prospective studies that evaluate temporal changes before and after surgery are warranted to further verify the clinical utility of PAI.

## Figures and Tables

**Table 1:** Patient demographics and treatment details.

Case	Age (Years)	BMI (kg/m^2^)	Type of Breast Surgery	Adjuvant Therapy	ASC Supplementation	No. of Grafting Sessions	Interval from Final Surgery to Imaging (Months)
1	54	27.1	SSM	Endo	No	3	26
2	59	21.5	TM	Endo	Yes	4	8
3	56	23.1	NSM	Chemo, Endo	Yes	6	18
4	47	20	NSM	Endo	Yes	7	3
5	55	20.8	TM	Chemo, Endo	No	3	12
6	67	26	SSM	Rad (pre-recon), Chemo	Yes	6	10
7	53	27.9	NSM	Endo	No	3	30
8	43	20	SSM	Endo	Yes	2	30

Abbreviations: ASC: adipose-derived stem cells; BMI: body mass index; Chemo: chemotherapy; Endo: endocrine therapy; NSM: nipple-sparing mastectomy; Rad: radiation therapy; SSM: skin-sparing mastectomy; TM: total mastectomy.

## Data Availability

The data presented in this study are available upon request from the corresponding author due to privacy and ethical restrictions.

## Abbreviations

The following abbreviations are used in this manuscript:

AFG	autologous fat grafting
ASCs	adipose-derived stem cells
BMI	body mass index
Chemo	chemotherapy
Endo	endocrine therapy
ICD	implantable cardioverter–defibrillator
NSM	nipple-sparing mastectomy
PAI	photoacoustic imaging
Rad	radiation therapy
ROI	region of interest
SSM	skin-sparing mastectomy
TM	total mastectomy
